# Cognitive performance of children living in endemic areas for *Plasmodium vivax*

**DOI:** 10.1186/s12936-017-2026-2

**Published:** 2017-09-12

**Authors:** Laélia M. B. F. Brasil, José L. F. Vieira, Eliete C. Araújo, Pedro P. F. Piani, Rosa M. Dias, Ana M. R. S. Ventura, Bianca C. Cabral, Renée C. R. Santa Brígida, Marcieni A. de Andrade

**Affiliations:** 1 Federal University of Pará (Universidade Federal do Pará-UFPA), Augusto Correa Street, 01, Belém, Pará 66075-110 Brazil; 2 000 0004 0620 4442 Evandro Chagas Institute (IEC), Rod. Br 316, Km 07, Ananindeua, Pará 67030-000 Brazil

**Keywords:** Malaria, *Plasmodium vivax*, Cognition, Intelligence scales, Tropical disease

## Abstract

**Background:**

The role of repeated episodes of malaria on the cognitive development of children is a relevant issue in endemic areas since it can have a long-lasting impact on individual lifespan. The aim of the current paper was to investigate whether the history of malaria can impair the verbal and performance skills of children living in an endemic area with low transmission of *Plasmodium vivax* malaria.

**Methods:**

A cross-sectional study was conducted with children living in an endemic area of *P. vivax* malaria in Brazilian Amazon basin. The history of episodes of malaria was used as criteria for inclusion of children in the groups. The cognitive performance was assessed by the Wechsler intelligence scale for children-III edition (WISC-III), which was applied to the participants of study by two trained psychologists.

**Results:**

A total of 17 cases and 26 controls was included in the study. A significant low score of verbal quotient was found in the cases (p = 0.005), however, the performance IQ was similar in both groups (p = 0.304). The full-scale IQ was significantly lower in the cases when compared to the controls (p = 0.042). The factorials index showed significant difference only in the subtest of verbal comprehension with the lower values in the cases (p = 0.0382), compared to the controls. The perceptual organization (p = 0.363), freedom from distractability (p = 0.180) and processing speed (p = 0.132) were similar in both groups.

**Conclusions:**

Children with a history of vivax malaria has a significant impairment of verbal and full-scale quotients as well as a significant low index of verbal comprehension. These findings are likely due to the absenteeism caused by malaria and by the low parental education, which impairs an adequate response to the environmental stimulus.

## Background

Malaria is still an important public health problem in several tropical countries [1]. In Brazilian Amazon basin, approximately 135,000 cases are reported each year and *Plasmodium vivax* is the predominant species accounting for 85% of reported cases. The disease affects equally all ages groups and both genders, and children are considered at risk to develop more serious signals and symptoms due to their relative lack of adequate immune response [2–4]. In fact, adverse effects on immune and haematopoietic systems as well as on the nutritional status have been described in this age group [3–5].

A relevant issue is the detrimental effect of repeated episodes of malaria on the cognitive development, which can have a long-lasting impact on individual lifespan. In fact, studies have demonstrated a lasting effect on cognition, behaviour and scholar performance of children with history of the disease [6–9]. Most of these studies were performed in patients with cerebral impairment or severe falciparum malaria, but it is likely that there is cognitive impairment in less severe infection including asymptomatic cases as well as in the short and long-term after the infection [8–12].

Different tools have been used to evaluate the cognitive impairment of children with malaria, some of them based on psychometric scales, which provide more reliable estimation of the impact of the disease on cognition [11]. There are several validated tests to assess cognitive performance, amongst them, the Wechsler intelligence scale for children-III edition (WISC-III), which is the most used scale to assess the intelligence as well as the degrees of neurological and psychiatric impairment in several conditions. The test also gives scores in healthy population about a variety of abilities associated with school success as well as it is considered one of the best predictors of future achievement [12–15].

In endemic areas, the findings of the cognitive performance tests provide rationale base for planning strategies of rehabilitation programmes, however, only a few studies assessed the cognitive impairment of children outside Africa [11]. For this reason, the objective of the current paper was to investigate whether malaria experienced by children from an endemic area from Brazilian Amazon basic with a sustained transmission of *P. vivax* can impair the cognition. The verbal and performance subtests scores of the Wechsler intelligence scale for children-III were measured in children with previous episodes of malaria and then compared to those of a control group with no history of infection by *Plasmodium*.

## Methods

### Ethical statement

The study was submitted to Plataforma Brasil under protocol CAAE 2 07199612.0.0000.0018 and approved by the ethical committee of Health Science Institute of the Para Federal University under the number 261.593/2013.

### Study area and participants

This was a cross-sectional study carried out at the municipality of Anajas in the Marajo Island (00º 59′ 21″S and 49º 56′ 24″W). Anajas has an area of 6912 km^2^ and 27,540 inhabitants, of which, 7347 children aged 2–10. The economy is based on agriculture practices, timber extraction, and fishing. The municipality accounts to 17.4% of malaria cases in the state of Para and has a constant annual incidence of cases above 50/1.000 inhabitants. Most cases (86.3%) are caused by *P. vivax*. The municipality has seven health public facilities, including an ambulatory for the diagnosis and treatment of malaria.

Children were randomly recruited in two schools of the municipality from January to December, 2014. The guardians of all children gave a written consent to their inclusion in the study and to collect a capillary blood sample for microscopic examination as well as answered a socio-demographic questionnaire. The cases were defined as children with negative diagnoses of malaria after microscopic examination of three blood smears for three consecutive days and with reports by the guardians of previous infection by *P. vivax*. The controls were defined as children with negative diagnoses of malaria after microscopic examination of three blood smears for three consecutive days and with no reports by the guardians of previous infection by *P. vivax* or *Plasmodium falciparum.* Exclusion criteria for both groups included: children with chronic and other parasitic diseases, obesity and underweight assessed by the body mass index, anaemia, congenital deficit, history of malaria in the last 6 months, and neurological or motor disorders. An additional criterion of exclusion in cases was the occurrence of neurological impairment in the previous episodes of malaria.

### Microscopic examination

Parasite counts were performed in Giemsa-stained thick films for three consecutive days. Blood films were examined microscopically using 100× (oil immersion) objectives. Parasite density was expressed as the number of parasites per microliter of blood. This was derived from the number of parasites per 200 white blood cells in a thick film, considering a total white blood cell count of 8000. The limit of detection of parasites was 40/µL [16].

### Data collection

Data were collected by two psychologists trained to administer and score the WISC-III using a set of test materials that require approximately 90 min to complete as well as the use of informed consent forms and the socio-demographic questionnaire.

### Socio-demographic questionnaire

A socio-demographic questionnaire was applied for all guardians of participants of the study, with questions concerning the area of residence (urban or rural), age ranges, scholar degree (fundamental, intermediary or superior), gender, familiar stipend, the history of malaria, considering the number of episodes and the time, in months, of the last episode of the disease and the inclusion in the study.

### Application of Wechsler intelligence scale for children-III edition (WISC-III)

The WISC-III was validated in Brazil in 2001 with 801 children from an urban area of the city of Pelotas, RS, with satisfactory values of convergence and discrepancy [17, 18]. The questionnaire has two batteries of subtests grouped into two general areas that are applied alternatively: (a) verbal subtests; and (b) performance subtests. The first evaluates the general knowledge, language, reason, and memory skills. Performance subtests measures spatial, sequencing, and problem-solving skills. The verbal subtests scores are summed and gave the verbal IQ score (VIQ); the same is done for the performance subtests scores which yield the performance IQ score (PIQ). Finally, the verbal and performance scores are summed and converted to the full-score IQ (FIQ). Scale scores may be converted to a quantitative scale, as follows: full scale scores beyond 130 places an individual in the superior or “gifted” range. Values between 111 and 120 are classified as high average range, between 90 and 110 as average; 80–89 as low average, 70–79 as borderline mental functioning and below 69 as extremely low [13, 17].

Furthermore, the WISC-III provides four factorial indexes derived from the verbal and performance subtests, which includes verbal comprehension (information, similarities, vocabulary, and comprehension), perceptual organization (picture completion, picture arrangement, block design, object assembly and mazes), freedom from distractibility (arithmetic and digit span) and processing speed (coding and symbol search). The indexes score also have means of 100 and standard deviations of 15. These factorial indexes provide complementary information to the individual set of subtests [13, 17, 18].

### Data analyses

Data are presented as frequencies of occurrence and as a mean and standard deviation or as median and range. Chi square test, Fischer exact test, and G test were used to compare proportions between case and control groups. Mann–Whitney U-test and Student *t* test were used to compare IQ scores and index scales between the groups of study. Data were analyzed using SPSS software, Release 21 (IBM inc, Chicago, IL, EUA). The significance level accepted was 5%.

## Results

A total of 17 cases and 26 controls met the criteria for inclusion in the study. The mean age of both groups was 8 years. The distribution of gender was similar in both groups (p = 0.19), but male predominates in cases and female in controls. The area of residence was similar in both groups with most of children living in the urban area. The mothers were the main guardians of children in both groups (p = 0.119) There is a significant difference in scholar degree of guardians with the prevalence of intermediary degree in the controls (p = 0.011), but the familiar stipend was similar in both groups (p = 0.127) (Table 1). The mean time from the last episode of disease to the enrollment in the study was 6 months. All cases were diagnosed and treated for *P. vivax* with the standard treatment recommended by World Health Organization [19].

**Table 1:** Baseline characteristic of children

Characteristic	Case (n = 17)		Control (n = 26)		p value
	n	%	n	%	
Area of residence					0.805
Urban	13	76	19	73	
Rural	4	24	6	27	
Gender					0.190
Male	10	59	10	38	
Female	7	41	16	62	
Previous episodes of malaria^a^					<0.001
1	0	0			
2	3	17			
3	3	17			
>4	11	66			
Last episode of malaria, months^b^	16 (5)				
Guardian					
Mother	16	94	19	73	0.119
Others	1	6	7	27	
Guardian scholar degree					
Fundamental	14	82	10	38	0.011
Intermediary	3	18	16	46	
Familiar stipend, dollars					0.127
<100	6	35	4	15	
>100	11	65	22	85	

^a^Number of previous episodes of malaria vivax reported by the guardian of children

^b^Interval between the last episode of malaria and the inclusion in the study

**Fig. 1:**
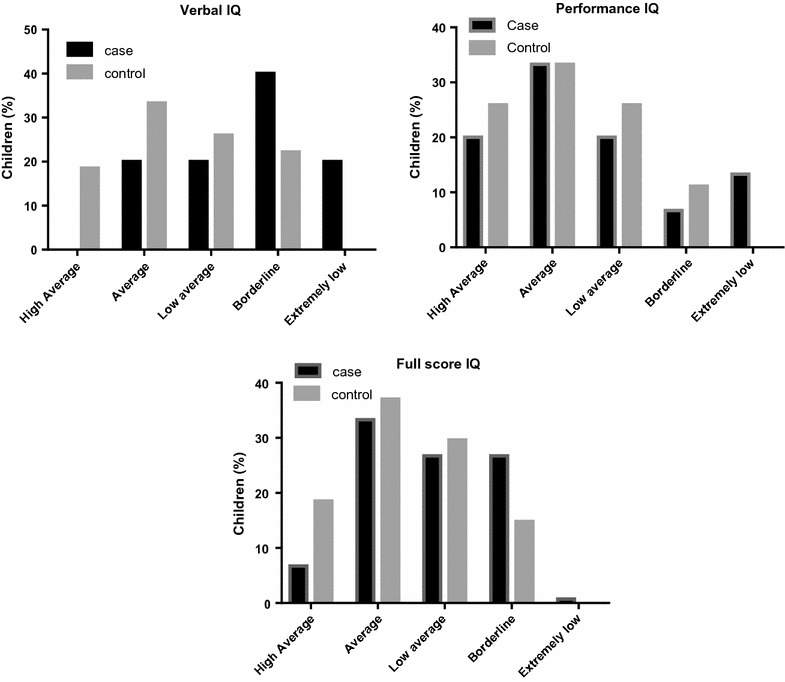
Qualitative classification of verbal, performance, and full-scale IQs in cases and controls

The scores of WISC III revealed a significantly lower value of the VIQ in the cases, compared to the controls (p = 0.005), however, the PIQ was similar in both groups (p = 0.304). The FIQ was significantly lower in the cases, compared to the controls (p = 0.042) (Table 2). The conversion of scores in qualitative scale revealed that most of the children of both groups have PIV and FIQs scores on the averages, and VIQs scores on borderline in cases and on the average in controls (Fig. 1).

**Table 2:** IQs scores of cases and controls

IQ	Case (n = 17)	Control (n = 26)	p value
Verbal	81 (12)	94 (16)	0.005
Performance	95 (21)	98 (14)	0.304
Full-scale	86 (15)	95 (15)	0.042

Results are express as mean and standard deviation

The factorials index showed significant difference only in the subtest of verbal comprehension with the lower values in the cases (p = 0.0382), compared to the controls. The perceptual organization (p = 0.363), freedom from distractability (p = 0.180) and processing speed (p = 0.132) were similar in both groups (Table 3).

**Table 3:** Scores of factorials index of subtests of cases and control

Factorial index	Cases (n = 17)	Control (n = 26)	p value
Verbal comprehension	81 (9)	91 (18)	0.0382
Perceptual organization	98 (22)	100 (18)	0.3639
Freedom from distractability	86 (23)	92 (15)	0.1809
Processing speed	85 (13)	89 (11)	0.1326

Results are express as mean and standard deviation

## Discussion

The study was designed to evaluate the cognitive performance of children from an endemic area of the Brazilian Amazon basin, where the transmission of *P. vivax* is sustained over the year. Overall, the disease courses with a low parasitaemia and mild signals and symptoms. Frequent and repeated episodes of malaria in children are common in this setting because they are continuously at risk of *Anopheles* bites. In the casuistic, male predominates in the cases and female in the controls, which may be due to the preference of male children for outdoor recreational activities which generally occurs in the proximity of *Anopheles* breeding sites. Most of the cases presented more than five episodes of disease with about one episode each year as well as there are no reports of cerebral impairment or other complications of the disease in all cases. The mothers were the main guardians of children in both groups as well as the familiar stipend was similar between groups, but the guardians of children from the control group have a higher scholar degree compared to the cases.

The instrument used to evaluate cognitive performance is recognized and used worldwide as a valuable tool to assess several aspects of intelligence based on the definition of Wechsler, that consider intelligence as an individual ability to adapt and constructively solve problems in the environment, that is, intelligence is understood as performance, not as capacity. Therefore, as intellectual capacity cannot be seen nor its existence concretely verified, it cannot be reliably measured; however, the performance may be evaluated. This finding is in line with most of the intelligence tests based on performance measures, such as the Stanford-Binet, the Peabody Picture Vocabulary Test, and the Guilford Intelligence Scales, however, Wechsler scale provide more a complete evaluation of the cognitive performance [14, 17, 18, 20].

The main risk factors of cognitive impairment in children living in Brazilian Amazon basin are anaemia, undernutrition, infestation by parasites and the exposure to environmental pollutants as mercury and pesticides [21]. Amongst these potential risk factors, only the exposure to environmental pollutant was not assessed, which can constitute the main limitation of the present study.

The scores of WISC III revealed significant low VIQ and FIQ in the cases compared to the controls. On the other hand, PIQ were similar in both groups. It is likely that the significant low score of FIQ of the cases was due to the low VIQ saw in this group because FIQ estimation is derived from the sum of VIQ and PIQ scores [13, 14]. The scores of IQs transformed in qualitative scale showed similar frequencies of distribution between cases and controls. Most of the children of both groups have PIQ and FIQ scores on the averages, and VIQ scores on borderline in cases and on the average in controls.

Large discrepancies between VIQ and PIQ have been reported in several diseases [18]. In the study, the differences between VIQ and PIQ was 14 in the cases and 4 in the controls. Thus, these differences validate the use of FIQ, since that only values above 17 decrease the importance of FIQ as an index to evaluate the level of intelligence performance of children [20].

The verbal and performance subtests used to determine the four factorials index revealed a significant difference only in verbal comprehension index with values significantly low in the cases compared to the controls. The other factorial index, as the perceptual organization, freedom from distractibility and processing speed were similar in both groups.

The VIQ has a high correlation with verbal abilities measures while PIQ correlates with nonverbal ones [13, 14]. Moreover, the subtests used to calculate verbal comprehension are closely related to verbal abilities. The VIQ refers to the language skill and general culture of an individual and has been considered a good predictor of school achievement. A low VIQ reflects a probable difficult to express the intelligence with words in response to environmental stimulation, that is, a difficulty for retaining verbal knowledge and comprehension from formal education as well as the application, by the children, of these abilities in new situations, which have been associated with a poor education, lack of educational and cultural opportunities, poor verbal skills, lack of environmental stimulation as well as a physical problem such as hearing or visual impairment [13, 14, 22–24].

In the study, the low scores of VIQ in the cases are likely due to the decrease of environmental stimulation that impairs an adequate response to this stimulus, resulting from both low parental education and scholar absenteeism [10–12, 15, 24]. The former is in line with a study that found a significant association between verbal abilities of children with parental education. The behaviour of the relatives has a significant impact on the cognitive development of children aged 6–9 [15]. Secondly, the repeated episodes of the disease contribute to school absenteeism as well as impose difficulties for interaction with relatives and children of the same age. Repeated episodes of malaria are accompanied by periods of treatment and of recuperation, which depends on the general conditions of children including the nutritional status, co-morbidities, and anaemia. Overall, a mean time of 30 days of absenteeism may be experienced by children from Amazon basin in each episode of malaria [3, 4, 11]. The latter is in line with a study in children from Sri Lanka in which the school absenteeism due to malaria was a significant predictor of cognitive performance as well as with studies that found a deficit in language abilities and low performance scholar in children with history of malaria. Finally, other extrinsic cause should influence the verbal abilities of children such as children–teacher relationship, the methodology of the teacher as well as the parental relationship [6–9, 11].

On the other hand, the data disagrees with the deficit in arithmetic reported in a previous study in the same area [11], since the freedom from distractability factorial index was similar in both groups. If these discrepancies are true, an influence of the tests used to calculate the freedom from distractability index or results from different tool to assess arithmetic skills, it should be further addressed.

The limitation of the study was the small number of children enrolled in both groups. Several efforts were done to increase the number of participants, however, the high incidence of the disease in the municipality of Anajas imposed difficulties to find children with no history of malaria. However, the data were similar to most studies that evaluated cognitive abnormalities in children with malaria, which can be due to the sensitivity of the Wechsler scale to detect minor change in the cognitive performance in this age group.

Finally, the data of the study are relevant to assist public health professionals in the planning of interventions to improve the verbal abilities of children living in endemic areas of malaria.

## Conclusions

Children with history of malaria by *P. vivax* has a significant impairment of verbal and full-scale quotients as well as a significant low index of verbal comprehension. These findings are likely due to the absenteeism caused by malaria and to the low parental education, which impairs an adequate response to the environmental stimulus.
